# A lineage explanation of human normative guidance: the coadaptive model of instrumental rationality and shared intentionality

**DOI:** 10.1007/s11229-022-03925-2

**Published:** 2022-11-21

**Authors:** Ivan Gonzalez-Cabrera

**Affiliations:** 1 Institute of Philosophy, University of Bern, Länggassstrasse 49, 3012 Bern, Switzerland; 2 Department of Psychology, University of Konstanz, Konstanz, Germany

**Keywords:** Coadaptation, Conceptual space, Lineage explanation, Goal-directed behavioral control, Reinforcement learning, Social norm psychology

## Abstract

This paper aims to contribute to the existing literature on normative cognition by providing a lineage explanation of human social norm psychology. This approach builds upon theories of goal-directed behavioral control in the reinforcement learning and control literature, arguing that this form of control defines an important class of intentional normative mental states that are instrumental in nature. I defend the view that great ape capacities for instrumental reasoning and our capacity (or family of capacities) for shared intentionality coadapted to each other and argue that the evolution of this capacity has allowed the representation of social norms and the emergence of our capacity for normative guidance.

A prominent view of the origins of morality focuses on the evolutionary and developmental interaction between instrumental rationality and shared intentionality (Gonzalez-Cabrera, 2017; Tomasello, 2016, 2020). On this view, hominin capacities for shared intentionality transformed the normative mental states supporting individual instrumental deliberation to facilitate the emergence of moral thought. In this paper, rather than provide a general justification for applying the shared intentionality framework to normative cognition, I expand on this approach to address the broader phenomenon of human social norm psychology (for a similar suggestion, see Gonzalez-Cabrera, 2017). The view put forward here aims to contribute to this literature by providing a more detailed account of (i) social norm representation, (ii) the way they could be algorithmically implemented in human cognition, and (iii) their coadaptive dynamics (see Glossary).

This view takes the form of a *lineage explanation* (Calcott, 2009), which aims to provide a tentative sequence of changes that makes increasingly plausible the emergence of social norm representation from a baseline of preexisting mechanisms within the hominin lineage. Building upon previous views of human norm psychology (Sripada & Stich, 2007), I characterize the *representation of social norms* as normative mental states that are defined by a gradient of *generalizability*, *intrinsic motivation*, and *corrective attitudes*. I argue that goal-directed behavioral control in the reinforcement learning and control literature defines an important class of intentional normative mental states, within which the representation of social norms is a special subclass. Following Tomasello (2016, 2020) and Gonzalez-Cabrera (2017), I claim that our capacity to represent and execute social norms was the result of the coadaptation of phylogenetically old capacities for instrumental reasoning in our great ape lineage and evolutionarily more recent skills for shared intentionality, which supported the representation and execution of commonly held social norms in humans.

The rest of the paper is divided into three parts. Section 1 discusses some conceptual issues regarding social norm representation. Section 2 focuses on cognitive architecture more deeply and review some of the features commonly ascribed to goal-directed behavioral controllers in the reinforcement learning and control literature. In it, I argue that this form of control can help us to single out an important class of intentional normative mental states closely linked to a phylogenetically old capacity for instrumental reasoning. Section 3 deals with the coadaptive dynamics. Here, I argue that the capacity of normative guidance requires some robust skills for shared intentionality and defend the view that great ape capacities for instrumental reasoning and our capacities for shared intentionality coadapted to each other to facilitate the representation of social norms and the emergence of our capacity for normative guidance.

## Social norm representation

**Fig. 1:**
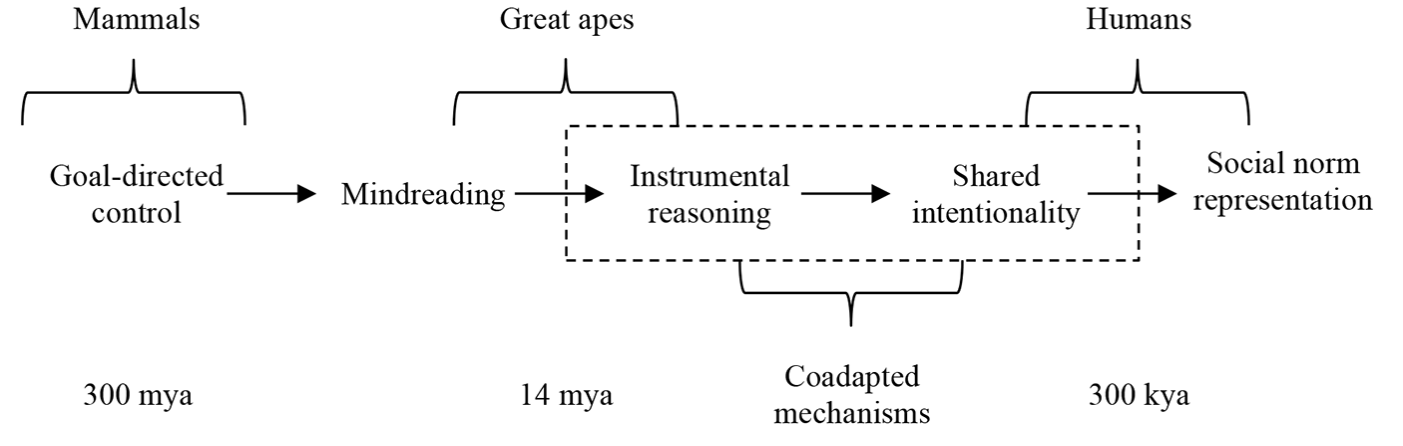
Proposed lineage explanation Note: Proposed lineage explanation of human social norm representation. The key sequence of traits (goal-directed control, mindreading, instrumental reasoning, shared intentionality, social norm representation) are divided according to the main lineage where they likely began to emerge (mammals, great apes, humans). Traits shown within dashed lines are coadapted mechanisms, which are the focus of the paper. Coadaptation occurs when interacting traits (instrumental reasoning, shared intentionality) undergo natural selection together in response to the same selective pressure or when selective pressures alter at least one of the traits, changing or creating an interactive feature (social norm representation).

Lineage explanations require a characterization of the state transitions in phenotypic space from an initial state to an end state. This section provides a characterization of human social norm representation as end state of the proposed lineage explanation. My goal here is making explicit the dimensions on which human normative guidance is supposed to be distinctive from great ape normative cognition (see Fig. 1).

Although social norms play a central role in different aspects of human life, most evolutionary views of normative cognition have particularly focused on the evolution of moral thought (e.g., Boehm, 2012; Joyce, 2006; Kitcher, 2011; Sterelny & Fraser, 2016; Stich, 2020; Tomasello, 2016; Wrangham, 2019). This is partially due to the vagueness of the term ‘social norms.’ These are often understood as the product of individuals’ interactions that solve collective action problems in the form of equilibria in game-theoretic approaches (Binmore, 1994; Gintis, 2009; Lewis, 1969; Young, 1998). As such, they are social level phenomena and their evolutionary dynamic has been fairly studied (e.g., Boyd & Richerson, 1992, 2002; Fehr & Fischbacher, 2004; McElreath et al., 2003; Ostrom, 2000; von Rohr et al., 2011; Yu et al., 2016). However, I will take social norms to be also a psychological phenomenon, i.e., a kind of mental structure that represents the aforementioned social phenomenon.

The representational function of social norm representations is twofold. When an agent represents a social norm, the agent could represent it as the solution to a collective dilemma that a social group actually follows (e.g., in the form of a population mean about what is customary to wear at a special ceremony or what resources are widely shared by all in the camp) but also what the agent thinks the group should do to solve such a dilemma (e.g., in the form of a hypothetical optimal solution about what the agent thinks they should wear at such events or the resources they should share with others in the camp).^1^ Hereafter, I will refer to social norms as these psychological phenomena unless otherwise specified.

Moreover, I understand the representation of social norms as singling out the class of normative mental states that provides *normative guidance*—our distinctive capacity to represent, endorse, and enforce social norms (for a similar formulation, see Kitcher, 2011). Since this class of normative mental states is conceived as being motivating and action-guiding, rather than merely fulfilling a representational function, the proposed analysis focuses on the representation of normative states that we *genuinely* embrace, as opposed to purely prudential or convenient extrinsic reasons.^2^ More specifically, this class of representations can be characterized in terms of three fundamental property gradients. The relevance of these gradients for the proposed lineage stems from the alleged differences in normative cognition between the ape and human lineages.

*Generalizability.* This gradient defines the scope of the normative state as conceived by the agent, from narrow- to wide-scope norms. A social norm is more general in scope the more individuals are represented as being able to fall within the scope of the norm. For example, developmental evidence indicates that people often distinguish moral from conventional norms because the former are universal while the latter are not (Nucci & Turiel, 1978; Smetana, 1981; Turiel, 1983). Norms that govern division of labor and gender roles apply to a group of people under certain generic social conditions. Thus, marriage rituals can be represented as having narrow scope because they apply to few individuals under specific conditions. Norms forbidding hurting innocent bystanders can, in contrast, be represented as having a wider scope. Although this aspect of norm representation is hard to test in nonhuman species, alleged normative behavior in chimpanzees seem highly constrained on the generalizability axis, such as when migrating females adopt the specific nut-cracking technique of their adoptive group (Luncz et al., 2015; Luncz & Boesch, 2014). They would be parochial norms. Thus, if chimpanzees represent social norms, these norms would fail to scale on the generalizability gradient.

*Intrinsic motivation.* This gradient defines the agent’s sensitivity to the content of the normative state, from merely instrumentally motivated to purely intrinsically motivated norms. A normative mental state is intrinsically motivating when the agent is motivated to comply, and make others comply, with that state as an ultimate end, rather than as an instrumental end. One may have different reasons to endorse ritual norms (e.g., fear of punishment), but sincerely endorsing those norms requires that we find their compliance rewarding in itself. Social norms can also be followed (though not genuinely endorsed) for purely instrumental reasons. Most likely, motivation is a mixture of intrinsic and extrinsic, instrumental reasons.^3^ Although these two can be seen as orthogonal dimensions rather than two diametrically opposed extremes of a single dimension, a gradient of intrinsic motivation can be operationalized, for the sake of simplicity, in terms of the sensitivity (e.g., the conditional probability) of norm compliance to extrinsic reward. For example, Bicchieri (2006, 2017) argues that individual norm compliance depends on a combination of punishment and a person’s sensitivity to the norm. For her, social norms are characterized by the fact that whether we follow them is conditional on what others who matter to us do and/or approve of. Moral norms, in contrast, are often considered unconditional. In the developmental literature, moral norms are often distinguished from conventional ones in terms of their independence of authority (Nucci & Turiel, 1978; Smetana, 1981; Turiel, 1983). However, cases of alleged normative compliance (moral or otherwise) in our closest relatives are more contested. For example, although longer looking times in chimpanzees at videos of infanticide than at control videos of aggressive displays toward adults have been suggested as evidence of a precursor of moral norms (von Rohr et al., 2015), they seem better described as curiosity-driven responses since, with the exception of one individual in this study, the stimuli did not reliably elicit negative emotional arousal in the observers. Similarly, since chimpanzees tend to copy the behavior of influential group members (Biro et al., 2003; Boesch, 2012; Horner et al., 2010; Kendal et al., 2015), alleged normative behaviors such as local nut-cracking techniques could be explained are more parsimoniously explained without appealing to social norms (Schlingloff & Moore, 2019). Human normative motivation seems distinctively robust.

*Corrective attitudes.* This gradient defines the agent’s degree of tolerance of the normative state against deviations, from low to high tolerance. A normative mental state engenders a corrective attitude if the agent is motivated to police, punish, or correct others, including themselves, when they think that they have violated the normative state. Corrective attitudes include, but are not limited to, clear-cut cases of punitive sanctions. Dancing or dressing inappropriately during a ceremony can trigger derision, scorn, and punishment, but also invites behaviors aimed at correcting the mistakes and promoting the ‘right way to behave.’ Developmental evidence, for instance, shows that moral transgressions tend to be judged more severely than transgressions of prototypical conventional norms (Nucci & Turiel, 1978; Smetana, 1981; Turiel, 1983; for a meta-analysis of the experimental evidence, see Yoo & Smetana, 2022). During conventional games, children often try to alter a transgressor’s behavior by teaching others the right way to play the game (Rakoczy et al., 2008). More importantly, deviations of social norms often trigger costly punishment by third-parties as widely reported in the experimental economics literature (e.g., Fehr & Fischbacher, 2004; House et al., 2020; Mathew & Boyd, 2014). And while great apes seem to punish others out of spite, evidence of third-party punishment is elusive (Riedl et al., 2012). Human investment in corrective interventions is distinctively costly.

**Fig. 2:**
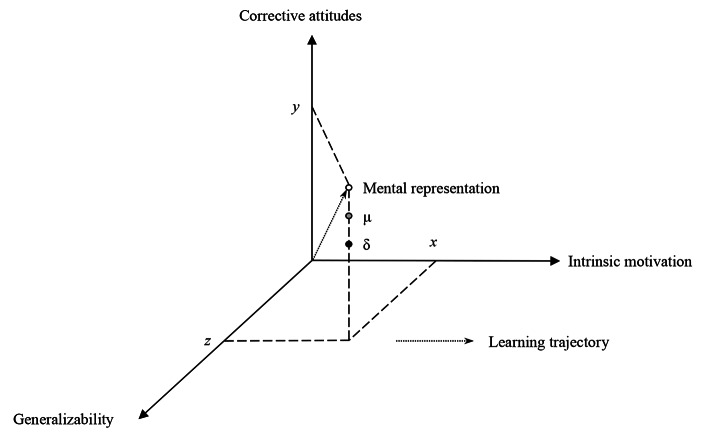
Three-dimensional representation of social norms Note: Each axis represents one of the property gradients previously discussed. An agent can represent a social norm as a point or vector in the three-dimensional space, which is defined by its coordinates along the axes. The doted arrow represents an agent’s hypothetical learning trajectory starting from the origin. Since social norms are represented through their parametrization over these gradients, such norms could be learned through policy gradient reinforcement learning algorithms, as suggested in Sect. 2.1. An agent can misrepresent a social norm by wrongly estimating its location with respect to either a population mean (µ) or a hypothetical optimal solution (δ)

The above characterization of social norm representation is inspired by that of Sripada & Stich (2007), while incorporating insights from a conceptual space approach, similar to that proposed by Gärdenfors (2000). On the analysis here, gradients are a set of separable quality dimensions that define a space in which social norms are assigned to individual points or vectors in such a space (see Fig. 2).

These dimensions are separable because we can theoretically assign objects (including social norms) a value in one dimension without giving it a value in another. Separability helps us to identify limit cases at the extreme ends of these dimensions. We might be skeptical of whether we can truly represent universal norms or empty norms such that no individual falls within their scope. Other limit cases may include norms whose motivational force is merely instrumental or purely intrinsically motivated. In the same way, we could argue that norms always trigger some kind of corrective attitude or that they always exhibit some degree of tolerance towards deviations. Some regions in conceptual space might be empty. Conversely, points in space could be correlated in interesting ways, as in the social domain theory in which moral and conventional norms cluster together around a set of well-defined properties (Nucci & Turiel, 1978; Smetana, 1981; Turiel, 1983; for critical discussion, see Kelly et al., 2007).

In this space, we can define a qualitative relation of nearness or neighborhood between points, even if no Euclidean metric can be defined for them.^4^ The more general a norm is represented as being, the higher its value in the generalizability-coordinate of the axis. The representation of a norm can also vary along a gradient of intrinsic motivation as a function of the motivational force they have on the agent. They can also vary along a gradient of corrective attitudes as a function of the tolerance towards the transgression of the norm. This helps not only to locate individual social norms in this space but also define nearness relationships between points, even if no uniform measure of distances can be provided. For the Hadza people in northern Tanzania, men that are able to hunt big game become *epeme* men. Only *epeme* men can eat certain cuts of these animals and perform the *epeme* dance performed on moonless nights (Marlowe, 2010; Woodburn, 1964). Thus, the Hadza’s representations of social norms, such as “Only *epeme* men can eat *epeme* meat” or “Only *epeme* men can perform the *epeme* dance,” could be located close to one another through their coordinate values in the resulting three-dimensional space.

This way to think about the representation of social norms is initially coarse-grained, so it might not be useful to draw fine-grain distinctions between social norms in the space so specified. But this is a desirable feature of the approach. It means that we can build finer-grained views of normative representations by adding up dimensions that correspond to more specific features of progressively narrower classes of norms. For example, one could add a gradient of explicitness where some norms are tacitly represented in the cognitive system, while others are more explicit and accessible to awareness, or even decompose the motivational axis into intrinsic and extrinsic component gradients. However, as dimensionality increases, the volume of the space increases exponentially. Thus, low-dimensional space can help us to see more easily how dimensions are correlated and how more general classes of norms partition the space. For example, according to the social domain theory, moral norms are universal, intrinsically motivated, and their transgression is judged more severely than other types of norm violations (Nucci & Turiel, 1978; Smetana, 1981; Turiel, 1983). On this view, moral norms are correlated across the proposed gradients. They define a region of vectors close to each other in conceptual space.

Moreover, the above three-dimensional space helps us to visualize how an agent can misrepresent a social norm in subjectivist and objectivist ways. The agent may misrepresent a social norm in a population subjectively by locating the norm in the wrong region of the space. For example, one can fail at locating a social convention such as “Only *epeme* men can eat *epeme* meat” by wrongly estimating the population mean µ (e.g., by overestimating the average degree of corrective attitudes that is associated with the violation of this norm in Hadza society as it appears in Fig. 2). Overestimation and underestimation might also happen along each of the axes of the theoretical three-dimensional conceptual space. For example, one may exaggerate the degree of generalization of the norm, or one may not be sufficiently motivated to follow it.

Somewhat differently, an agent may think that a social norm objectively represents the optimal solution to a certain decision-making problem. We represent not only the existing norms of social groups but also the social norms we think such groups should adopt under certain criteria. For example, a Hadza hunter may think that only *epeme* men should eat *epeme* meat because this prevents illness and death. Otherwise, he believes, consuming this meat causes serious illness or even death (Marlowe, 2010). As with other social norms, food taboos about *epeme* meat among the Hadza are decision-making mechanisms. An obligation to do a certain action such as eating *epeme* meat only when you are an *epeme* man, for instance, can be understood as a solution to the question of what to do in a certain situation. The decisions this obligation prescribes maximizes the agent’s perceived value of his/her action. But agents sometimes treat the ascribed value as an objective feature of the action.

The agent can fail at representing this solution for two reasons. First, the agent can wrongly think that there is a single optimal solution to the problem at hand when there is none, as one might suspect in the case of many normative aspects of *epeme* rituals in the Hadza. For example, a norm such as “Only *epeme* men can eat *epeme* meat” is likely not an optimal solution to the problem of meat distribution or public health among the Hadza. Second, assuming there is an optimal solution δ to the problem, the agent may fail at representing its location along some of the coordinates of a three-dimensional space of hypothetical solutions (e.g., along the gradient of corrective attitudes as shown in Fig. 2). Thus, an agent can misrepresent a social norm either because the agent fails to represent the location of a social norm in a population or because the agent fails at representing a social norm as a single optimal solution to a problem.

Do great apes represent social norms and are able to be guided by them in the way just described? Likely not. But even if they do, the conceptual space approach could help us to better understand the differences in degree rather than kind between our species normative dispositions. The view defended in the next section ascribes some baseline capacities in normative thought to great apes and so likely to our early hominin ancestors. But these baseline cognitive capacities facilitate normative thoughts and motivations that are too egocentric to be sufficiently general, intrinsically motivating, or capable of generating the kind of costly corrective attitudes that characterizes human social norm psychology.

## Goal-directed behavioral control

There are indeed alternative accounts of the evolution of social norm psychology, which do not exclusively focus on moral thought (e.g., Birch, 2021; Chudek & Henrich, 2011; Gavrilets & Richerson, 2017; Theriault et al., 2021). Such accounts, however, could benefit too from the kind of conceptual space approach of the representation of social norms just outlined. Furthermore, these evolutionary views typically avoid discussions of algorithmic implementation (although for a notable exception, see Birch, 2021; Theriault et al., 2021). Although mechanisms such as gossip, social disapproval, or ostracism create selection pressures for people to avoid punishment by learning what actions are considered permissible and creating an intrinsic motivation to comply, these models are not meant to be accounts of the algorithmic implementation involved in the process. This computational gap is even more evident in approaches based on instrumental rationality and shared intentionality, which often rely on comparative and developmental data (Gonzalez-Cabrera, 2017; Tomasello, 2016, 2020).

In the view I propose, goal-directed behavioral control in the reinforcement learning and control literature defines an important class of intentional normative mental states, within which the representation of social norms is a special subclass. Intentional mental states can be broadly described as normative when they are action-guiding. In this broad sense, many mental states are *proto*-normative because their biological function is to guide behavior toward the organism’s goal. This relation is not only phylogenetic but functional. To the extent that these states fulfil a similar functional role, they could count as having proto-normative content as per views of “inferential” or “functional” role semantics in philosophy and cognitive science—i.e., the idea that the content of a mental representation is at least partially constituted by the role they play in cognition (see, for instance, Block, 1986). In this sense, pushmi-pullyu representations in the teleosemantic literature (Millikan, 1996; Papineau, 1984; for discussion, see Shea, 2018) would count as proto-normative because they carry both descriptive and imperative content (e.g., simple internal commands such as “Seek shelter,” “Feed the young,” or “Fly from danger”).

This section shows how this body of research moves us one step closer to social norm representation. It does so by sketching the mechanisms by which we learn and execute intentional normative mental states (or ‘behavioral policies,’ as I will call them). These policies have not only descriptive content (i.e., they specify what the agent should do in a given situation) but also imperative content in light of the agent’s goals. As we will see, our ape-like mindreading capacities leveraged these policies to scaffold a form of individual instrumental reasoning (see Sect. 2.1). However, acquiring social norms requires further constraints on the representation of intentional normative mental states to generate a conceptual space of social norms as shown in Fig. 2 that helps us break the solipsistic curse of our ape-like normative mental states. This is the basic insight of shared intentionality approaches: shared intentionality meshes baseline normative mental states to support joint activity by extending these structures from a mostly individual perspective to a group or shared perspective. A coadaptive view of this process is offered in Sect. 3 based on the computational approach and incremental stages sketched in this section.

Computational mechanisms for learning and control are divided into *instrumental* and *non-instrumental* (see Table 1).

**Table 1:** Different systems for learning and control, and their distinctive features

	Systems of behavioral control			
Control system	Reactive	Pavlovian	Habitual	Goal-directed
Response	Fixed	Flexible	Flexible	Flexible
Contingency	None	*S*→*O*	*B*→*O*	*B*→*O*
Shaping process	Evolution	RL	RL	RL
Type of control	Non-instrumental	Non-instrumental	Instrumental	Instrumental
Computation	Model-free	Model-free	Model-free	Model-based via the representation of goals and *B*→*O* contingencies

*Note.* Reactive control provides fixed responses that are shaped by evolution, and thus are not sensitive to the contingent environmental relationship between stimuli (*S*), behaviors (*B*), and outcomes (*O*). Pavlovian, habitual, and goal-directed systems of behavioral control provide flexible responses that are shaped by reinforcement learning (*RL*) to be sensitive to environmental contingency. Since only habitual and goal-direct control systems are sensitive to the contingent relationship between behaviors and outcomes, only these systems provide instrumental forms of control. Goal-directed control is the only instrumental form of control that relies on a model-based computational approach that represents the goals of the agent and the contingent relationship between behaviors and outcomes

In instrumental control, behaviors are controlled via mechanisms that are acquired by learning the contingent relationship between actions and outcomes, i.e., by estimating the probability of an outcome given that the action is performed and the probability of the outcome given that the action is not performed (Hammond, 1980). This form of control is instrumental in the sense that the organism learns to produce the behaviors that are instrumental to achieving the desired outcome, rather than relying on non-instrumental *reactive* and *Pavlovian* forms of control. For example, salivation is a reactive response, shaped by evolution, to the presence of food in many animals. Dogs can salivate if they learn that the sound of a bell predicts food (Pavlov, 1927), as opposed to learning that salivation leads to a desired outcome, such as making the food more palatable. In contrast, instrumental control helps organisms to extend their response breadth by reinforcing new behaviors (e.g., pressing a lever) as a function of the desired outcome to which those responses lead (e.g., obtaining a food reward).

Instrumental control can be implemented via mechanisms of *habitual* behavioral control. These mechanisms allow the agent to maximize rewards and minimize punishments by learning the contingent relationship between actions and outcomes based upon previous experience and using information about past rewards and punishments to choose the best available course of action in a specific context. This course of action is described by a behavioral policy, which is a mapping from environmental states to the actions that are the best in those situations. Since these systems search for policies in a value function space without requiring a model of the environment’s transition dynamics, these systems are considered model-free. When an agent performs an action that leads to a reward, these mechanisms increase the value associated with the action. The value of the action decreases, in contrast, when it does not lead to the expected reward. Since decisions are made based on values stored in a cache memory, habitual control does not require computationally demanding inference. This form of control is fundamentally retrospective. When a change in the environment modifies the utility of an outcome, new cached values can only be acquired via direct experience. This means that this form of control is not immediately sensitive to reward devaluation.

However, mammals are also capable of goal-directed behavioral control, which depends on a model of the world that represents the contingent relationship between behaviors and outcomes, and the representation of the agent’s goal as a possible outcome (Dickinson, 1985). This is a flexible, reward-sensitive, but computationally intensive form of control. It is flexible because environmental changes that affect the expected utility of an action could be predicted by adjusting the organism’s model of the world. This makes this form of control more sensitive to reward devaluation than habitual control. It is computationally intensive, though, because policy search heavily relies on inference rather than memory. It requires agents that choose the suitable means to their ends. Their actions should maximize long-run rewards, or minimize long-run punishments, through inferential processes that execute a form of dynamic programming (Howard, 1960) or forward or backward searching (Foster & Wilson, 2006). For example, an organism such as a rat might solve an experimental task by forming a cognitive map of a maze and the location of the food reward on it. Using this map, the rat can then choose the behavioral policy that maximizes long-run rewards in policy space by inferring the best action to take at a particular point in the maze in order to get the reward (Dolan & Dayan, 2013).

Goal-directed behavioral controllers can be partially dissociated from all of the above forms of control not only psychologically, but also neurally and computationally (Balleine & O’Doherty, 2010; Dayan, 2012; Huys et al., 2011; Wunderlich et al., 2012). In what follows, I will set aside issues concerning neural implementation and focus on model-based approaches to reinforcing learning in computer science and engineering that describe goal-directed behavioral controllers. Leaving these details helps us to understand more easily the connection between these controllers and the representationally rich computations used in these approaches. For example, in more basic approaches, action effects and goals are given to the agent, while in Markov decision processes (which provide the framework of most reinforcement learning algorithms) they are learned. Once the agent has adequately modeled the environment and its goal, it can use its internal model to find the best course of action in policy space.

This is why these computational approaches to learning and control are considered model-based: decisions are made based upon an internal model of the agent’s goals and the contingent relationship between actions and outcomes, which is built through reinforcement learning processes. To the extent that model-based control systems provide a computational description of goal-directed forms of behavioral control, I will call the set of representations involved in these computational descriptions the ‘goal-directed controller’ (Dolan & Dayan, 2013).

### Goal-directed policies and instrumental reasoning

Some views of normative cognition are grounded in model-based forms of cognitive control. For example, Birch’s (2021) locates the emergence of this form of cognition in the context of model-based motor control and the transmission of toolmaking skills, a view that has been endorsed by others (e.g., Sterelny, 2021).^5^ In the view proposed here, however, a motor skill approach can be seen as a special case of a more general lineage explanation that links normative cognition to instrumental rationality and, more specifically, instrumental reasoning.^6^

As mentioned above, goal-directed controllers also allow us to define a special class of intentional mental states. The goal of this section is to explain how the behavioral policies that are the output of these controllers can be construed as intentional normative mental states that are intrinsically linked to a form of instrumental reasoning. By linking behavioral policies to instrumental reasoning, this transition takes us one step closer to social norm representation. For the gradient parameterization of Sect. 1 is supposed to operate on states that are already normative, such as the behavioral policies of instrumental reasoning, even if those states are not themselves generalizable, intrinsically motivating, or tied to corrective attitudes. Not all intentional normative states need to be parameterized in this way, but all states representing genuinely endorsed social norms need to be normative.

Unlike reactive, Pavlovian, or habitual responses, goal-directed choices depend upon representationally rich states that rely on a heavy intentional gloss (Egan, 2014). Conversely, these other systems show that an organism’s behavior can be controlled by very simple normative mental states, avoiding an overintellectualized picture of behavioral control. All these states are normative in the broad sense of being action-guiding mechanisms. Furthermore, model-free and model-based controllers can represent parameterized policies that further constrain policy space. Since policies can be parameterized in any way, so-called ‘policy gradient methods’ would allow states similar to social norms to be represented in the system by introducing the relevant parameters in conceptual space (see Fig. 2).

Policy gradient methods aim at modeling and optimizing the behavioral policies directly (Sutton et al., 2000). The agent learns these policies by optimizing the value of the parameters with respect to long-term cumulative reward, driving the agent’s learning trajectory contingent upon her socio-cultural environment. Importantly, although policy gradient methods can be model-free and model-based (D’Oro et al., 2020; Peters & Bagnell, 2016; Wang & Dietterich, 2003), only the latter class of policies would be normative in the narrower sense of guiding behavior given the agent’s *goal representations* and her *model* of the chain of transitions between environmental states. This makes these policies akin to hypothetical imperatives. A policy constitutes the representation of a genuine normative constraint that governs the agent’s action insofar as the agent must be committed to it as part of her own volitional activity as an agent. This view assumes that commitment to the actions that lead to the desired outcome is a necessary condition for volitional action, i.e., an agent should be committed to the means that are suitable for achieving her goals if the agent wants those goals at all. Since these states represent suitable means to achieve the agent’s goals, maximize long-run rewards, or minimize long-run punishments, they are subject to conditions of satisfaction. Some mental states will represent means that satisfy the agent’s goals, while others will not. Thus, failing to commit to the means needed to attain those goals would entail failures of volitional action since the agent would not be able to do what the agent wills to do.

Goal-directed controllers make organisms instrumentally rational. Once an organism has a model of the environment, decision-making is a matter of planning, i.e., the organism can compute a sequence of actions that optimizes the outcome reward before executing a decision. When organisms are able to compute this sequence of actions, they are instrumentally rational to the extent that they are able to interpose such actions as a set of states between a representation of their current circumstances and a goal state (Camp & Shupe, 2017). The organism’s internal model represents and executes a policy because such a policy is an achievable difference-maker for achieving a goal, as opposed to executing this sequence because it is intrinsically rewarding.

This does not mean that the organism has to be aware of the inner functioning of these goal-directed controllers. Goal-directed controllers *can* be subpersonal-level mechanisms and therefore inaccessible to consciousness.^7^ They rely on first-order representations of the organism’s goals and the transition between environmental states, but they do not require metarepresentational capacities that would bring these states above the level of awareness. Executive processes are often considered metacognitive because they monitor and control other cognitive processes, but only some metacognitive processes are metarepresentational in the sense that they involve self-directed metarepresentational states as opposed to other-directed metarepresentational states such as mindreading (Carruthers, 2014). Yet, unlike other forms of control, goal-directed controllers specify the structure and components of inference. This feature, then, likely helped our hominin ancestors to become increasingly aware of the relevant mechanisms—including the associated policies—which otherwise would remain inaccessible to consciousness. For mindreading practices could track the inner functioning of goal-directed controllers, even if imperfectly and in a piecemeal fashion, e.g., as if they were instances of some form of instrumental reasoning.^8^

Many mammals seem to lack higher-order representations of these goal-directed controllers and their outputs, such as beliefs about their own (or others) normative mental states. Great apes, in contrast, are characterized by some capacity for mindreading (Krupenye et al., 2016). This capacity arguably allows primate species such as the chimpanzee to have representations that track and respond to the intentional normative mental states of other individuals’ goal-directed controllers. This would allow chimpanzees, and perhaps other social but predominantly hierarchical species, to have higher-order representations of the imperative demands of alpha individuals in their groups. Moreover, by turning our mindreading capacities upon themselves, our ape ancestors would have been increasingly able to understand their own minds. The idea that metarepresentational capacities are the result of the elaboration of mindreading capacities in this way is not new (Carruthers, 2009). This metarepresentational capacities would have transformed mammal instrumental rationality into a more human-like form of explicit reasoning, as it is often reported in the ape cognition literature (Bohn et al., 2017; Völter et al., 2016; Völter & Call, 2017).^9^ For example, comparative research shows that great apes engage in more information seeking when they had no prior knowledge of food and tool items (Bohn et al., 2017) and they are capable to represent alternative possibilities and reason about what could be the case in controlled food retrieval tasks (Engelmann et al., 2021).

All the above features make policies in model-base controllers promising candidates as precursors of social norm representations in a lineage explanation of human social norm psychology. As explained in more detail in Sect. 3, in a lineage like ours where social coordination became increasingly important, awareness of one’s own and others’ behavioral policies reduces uncertainty, increasing the chances of successful social coordination. However, there are reasons to think that mindreading alone did not lead to the emergence of human capacities for normative guidance. The solipsistic curse of normative mental states does not go away so easily. Normative guidance requires not only the faithful transmission of the descriptive content of an intentional normative mental state but also that of the *attitudes* associated with its *genuine adoption*. Mindreading helps us to keep track and be aware of the content of such states, but this does not suffice for normative guidance. On the view advanced in this paper, normative agents would need not only to be aware of goal-directed policies (which would otherwise be purely subpersonal) but also to be able to share those policies with others.

### Social norms as shared goal-directed policies

Most views about the evolution of moral thought and normative cognition more generally accord little to no role to shared intentionality in their accounts. Certainly, a multitude of different types of mental structures might lead to compliance and enforcement behavior of the sort that allows many to identify the presence of normative behaviors (e.g., compliance with alpha-male commands). It is more difficult though to explain why we share with others our normative representations over and above simple instrumental reasons. For instance, one can imagine individuals internalizing food taboos and enforcing such behaviors driven by fear, coercion, or simple disgust. But the puzzle of normative guidance is to explain how we sincerely adopt social norms and why we want others to do so as well. Computationally, the best available models for this come from the reinforcement learning and control literature, which not only gives us a model for processing punishment signals but also reward signals. Shared intentionality views in turn help us to understand why we share social norms and why we join them sincerely by transforming punishment and reward signals. For the shared intentionality hypothesis states that although great apes attribute some mental states to others, they are not necessarily intrinsically motivated like humans to share those psychological states (Call, 2009).

Not all goal-directed policies represent social norms in the sense specified in Sect. 1 since social norm representation requires a particular form of parameterization over gradients of generalizability, intrinsic motivation, and corrective attitudes (see Fig. 2). In this section, I argue that in order to parameterize policies in this way, hominins needed to evolve a capacity (or family of capacities) for sharing those intentional normative mental states. In other words, normative guidance required a capacity for *shared intentionality* that not only allowed us to represent groups as intentional agents, but also to reliably engage in the intentional mental states that we ascribe to those groups, including the intentional normative mental state generated by our goal-directed controllers.^10^

There are theoretical reasons to think that behavioral policies that represent social norms are shared intentional mental states. For the representation of social norms are intentional mental states and these intentional mental states have to be represented as shared within a group of agents. The reasons for this are conceptual, but only partially so. For example, social norms are intentional in the sense that they are about the various aspects of our lives they aim to govern (e.g., gender roles, division of labor, sexual behavior, trade, and warfare). Like some policies, social norms are goal-directed; they have a purpose. The content of a representation of a social norm can, then, be roughly specified through a proposition that expresses some goal-directed policy toward which the agent takes a propositional attitude. We endorse these norms, we comply with them for different reasons, or we reject them. These mental states can misrepresent social norms in different ways. Our representations of social norms may fail at grasping the local mores and conventions of our social groups or the objective solution to a certain decision-making problem. The proposed three-dimensional space of Fig. 2 helps us precisely to visualize how an agent can misrepresent its normative environment. Thus, social norm representations in the form of goal-directed policies are intentional mental states.

Furthermore, if a policy π were not represented as a shared intentional mental state, then π would not be represented from a shared perspective or would not be the result of an intrinsic motivation to join or share those mental states.^11^ Indeed, shared intentional states are often described as having a *we-mode of representation* (Gallotti & Frith, 2013), entailing a *bird’s-eye point of view* (Fletcher et al., 2012), or being agent-neutral (Pacherie & Dokic, 2006; Rakoczy, 2017; Satne & Salice, 2020). This might be carried through different mechanisms. For example, Tomasello and colleagues have argued that this is sometimes achieved through recursive forms of mindreading (Grueneisen et al., 2015; Tomasello et al., 2012). Alternatively, shared intentional mental states could be represented through a we-mode of representation that is irreducible to mindreading. Recently, it has been argued that shared goals (Djalovski et al., 2021; Fishburn et al., 2018) and joint attention (Koike et al., 2016; Saito et al., 2010) involve a form of interpersonal neural synchronization that cannot be reduced to responses at the individual level. This form of neural encoding is sometimes considered a marker of shared intentionality (Barraza et al., 2020).

Henceforth, I will refer to mental states that have any of the above properties as having a *shared perspective*, since shared intentionality contributes to the generalizability gradient through a combination of such properties. Consistent with this, for example, if a goal-directed policy π were not represented from a shared perspective, the agent in question would not represent herself and others as entertaining π together, as in the thought “We ought to φ.”^12^ Many, though not all, social norms have precisely this form. Even quite narrow-scope norms such as the ones that govern reciprocity or ritual practices in clan-based societies (Flannery & Marcus, 2012) have, arguably, some degree of generalizability in this sense. Norms apply to individuals playing certain social roles in certain circumstances. More generally, representing social norms requires representing an intentional normative mental state π such that multiple subjects, including the agent herself, are able to fall within the scope of that policy. This could be so because the norms are agent-neutral, represented from a bird’s-eye point of view, or through a we-mode representation, as in the example above. Therefore, π could be represented from a shared perspective if π represents a social norm.

Alternatively, if a goal-directed policy π were not the result of an intrinsic motivation to join or share those mental states, then the agent in question would be neither intrinsically motivated to join π nor intrinsically motivated to share it. Assuming that the agent is not intrinsically motivated to join π means that the agent is, at most, motivated to be in a normative mental state π’ that mirrors the policy π of another for purely instrumental reasons. Likewise, if the agent is not intrinsically motivated to share π with others, the agent displays π, at most, to make another agent join that policy for purely instrumental reasons. But representing social norms that we genuinely follow requires an intrinsic motivation to comply, and to make others comply, with those norms. The agent must then not only represent π from a shared perspective but also be intrinsically motivated to join or share that policy, assuming that such policy represents a social norm. Therefore, social norm representations, including those represented via goal-directed policies, seem aptly construed as shared intentional mental states.

The underlying empirical assumption of the above view is that there are aspects of normative guidance that are uniquely human and thus absent in other apes and mammals. So we need an account of how they evolved in the hominin lineage. As the instrumental rationality approach suggests, this distinctiveness is largely driven by sincere endorsement over and above those instrumental reasons, suggesting that species differences are at least partially motivational rather than strictly cognitive.

There are only a handful of empirical studies that provide suggestive evidence of socially normative guidance in non-human animals. For example, as mentioned earlier, work on conformity to tool-use practices in chimpanzees shows that immigrant females abandon the nut-cracking technique of their natal group in favor of a sometimes less efficient technique practiced by their foster group (Luncz & Boesch, 2014). Similarly, work on animal play by Bekoff & Pierce (2009) and Flack et al., (2004) appears to suggest that play is governed by rules such as self-handicapping when playing with younger individuals or when and what play signals to give. However, these results are unable to rule out whether conformist transmission of nut-cracking technique is the result of copying dominant individuals or whether play behavior is merely the result of individual play preferences. For example, chimpanzees are known to copy the behavior of influential group members (Biro et al., 2003; Boesch, 2012; Horner et al., 2010; Kendal et al., 2015) and evidence in chimpanzees and bonobos indicates that they prefer to play individually rather than with a conspecific when given the chance (MacLean & Hare, 2013; Warneken et al., 2006). Attempts to reengage recalcitrant partners have been reported only when interacting with human partners who are supposed to possess the motivations and cognitive competences for shared intentionality—a behavior similarly observed in control conditions lacking triadic engagement (MacLean & Hare, 2013).^13^

Human normative guidance is not only cognitively complex but also motivationally demanding. Brosnan & de Waal (2003) have famously shown that brown capuchin monkeys refuse to participate if they witness a conspecific obtaining a more attractive reward for equal effort. However, protests only occur in cases of disadvantageous, but not advantageous, unequal distribution, suggesting that expectations do not extend toward third parties, as is expected in the case of social norms. Moreover, these findings could be explained by disappointment rather than social expectations on reward distribution since subsequent studies in monkeys and chimpanzees reveal that protests are directed only at the experimenter regardless of the presence of a social partner (Engelmann et al., 2017; Wynne, 2004).

Nonhuman animals are certainly capable of forming social expectations, or perhaps even norms, other than those specified in Sect. 1 (Andrews, 2009, 2012; for discussion, see Schlingloff & Moore, 2019). This is especially true for great apes, which for the purpose of the present lineage explanation is the relevant comparison class. But in that lineage there is little or no evidence of costly (altruistic) third-party punishment as in humans, even though *precursors* of social norm psychology such as nepotistic punishment, coalitionary retaliation, and dominance-driven policing do exist (von Rohr et al., 2011; von Rohr et al., 2012, 2015). Similarly, impartial intervention by third parties in ongoing conflicts is rather rare and consistent with the *group stability hypothesis* (Flack et al., 2005), which predicts that these behaviors are carried out by high-ranking individuals because they have the power to effectively stop aggression at a lower risk of retaliation. Although impartial interventions do not involve punishment and are not biologically altruistic (i.e., they bring net benefits to arbitrators by allowing individuals to maintain larger social networks and increase group stability and rank), they could indeed be motivated by some form of group concern.^14^ Nonetheless, the largest observational study showing altruistic third-party punishment in chimpanzees reveals that third-party outsiders intervened only 14 times out of 175 observations with only 4 being impartial (Suchak et al., 2016). Taken together, this evidence indicates that non-human primates likely lack the mechanisms for representing and executing shared social norms.

To sum up the argument so far, the focus of attention in this section changed from goal-directed policies to the sharing of those policies. Yet not all shared policies are social norms in the way specified in Sect. 1 since this requires a special type of parameterization. I argued, instead, that if a certain goal-directed policy π is the representation of a social norm, then π must be a shared normative intentional mental state. The reasons are partially conceptual, but empirical evidence also suggests that the sort of normative behavior in great apes is not the same as the one distinctive of humans.

## The coadaptation of instrumental reasoning and shared intentionality in the evolution of normative guidance

If behavioral policies represent social norms when they are shared normative intentional states, shared intentionality should explain the parameterization of these policies over the proposed gradients of generalization, intrinsic motivation, and corrective attitudes. On the view I propose, the evolution of a capacity for shared intentionality affected the developmental and evolutionary trajectory of preexisting capacities for instrumental reasoning in the hominin lineage by enabling agents to engage in means-end reasoning of the form “If we want *x*, we ought to φ.” This is so because capacities for shared intentionality allowed the agent to represent goals and intentions from a shared perspective such as “We want *x*.” These joint and shared goals would have subsequently served as inputs for preexisting mechanisms for instrumental reasoning. In other words, they transformed the policy search space of hominin goal-directed controllers by supporting forms of decision-making based on shared normative intentional mental states that facilitate the solution of collective dilemmas. Typical individual forms of instrumental reasoning such as “If I want *x*, I ought to φ” would have been extended to the social domain and transformed into a kind of *social instrumental reasoning* of the form “If we want *x*, we ought to φ,” i.e., a capacity to engage in a form of means-end reasoning in which joint or shared goals are the input of instrumental reasoning.

One reason why great apes are thought to lack capacities for shared intentionality is that they seem unable to form stable shared goals (Warneken et al., 2006). For example, when collaborative activities are disrupted by a suddenly uncooperative partner, chimpanzees (unlike human children from about 18 months of age) often do not attempt to reengage their partners and prefer to go solo when those partners are not necessary for the task. Reengagement efforts have been reported in the literature mostly from observational studies (Gómez, 2010; Pika & Zuberbühler, 2008; Tanner & Byrne, 2010; although see MacLean & Hare, 2013) but only when those efforts are low cost (e.g., when no third-party punishment is involved) and when they interact with human experimenters, which are highly competent and motivated social partners.

This is important because reengagement is a proxy of commitment that, in turn, stabilizes joint efforts when they are costly and need to be sustained over time. The same goes for social norms as they require agents to stick to them. Evidence of this form of commitment has been elusive in great apes (Greenberg et al., 2010) but not in young human children (Hamann et al., 2012). Alleged evidence of shared intentionality in great apes comes from studies reporting the resumption of social grooming and behaviors suggestive of reengagement efforts such as gestures and vocalizations after interruptions in controlled and natural settings (Genty et al., 2020; Goldsborough et al., 2022; Heesen et al., 2020, 2021, 2022). However, as in the case of alleged social norms, these results could be driven by individual preferences for social over nonsocial activities, rather than shared commitments that help partners to maintain costly cooperation. In many social mammals, social interaction is intrinsically rewarding (Panksepp et al., 1997; Trezza et al., 2010).

Even if the above examples count as cases of shared intentional states (albeit in some qualified sense), great apes would still lack the adaptations for share intentionality that allow humans to sustain these shared states over time.^15^ This would be crucial in the case of social norms as they are supposed to provide reliable behavioral guidance. In such a case, the evolution of human social norm psychology would be similar to explaining, say, bipedalism in the hominin lineage, where previously existing structures adjust to each other due to increasing selection for this form of locomotion (e.g., adaptations to maintain stability and save energy when standing, walking, and running; see Lieberman, 2014) while, for instance, dealing with gradual encephalization that leads to cephalopelvic disproportion (i.e., the mismatch between the fetal head and the mother’s pelvis; see Fischer & Mitteroecker, 2015; Washburn, 1960; Wells et al., 2012; Wittman & Wall, 2007).

A plausible hypothesis is then that mechanisms for instrumental reasoning as seen in great apes began to coadapt with skills for shared intentionality in the context of the hominin transition toward high-risk collective dilemmas such as big-game hunting and other forms of foraging that require organized defense, instead of conceiving hominin collective foraging as the primary driver of the evolution of shared intentionality as a whole (Tomasello et al., 2012). Coadaptation refers here to the mutual adaptation of parts within an organism, which require mutually adjusted changes in their components. Big-game hunting and other collectively risk forms of foraging required not only instrumental reasoning, but also the sharing of plans, beliefs, and goals among foragers to coordinate action in collective dilemma situations. There is good evidence that hominins were hunting antelopes (e.g., kudu and wildebeest) by 2 mya (Diez-Martín et al., 2016; Domínguez-Rodrigo, 2002). By the time power scavenging and hunting were part of their foraging spectrum, these activities would have been complex enough as to require organized collective action, creating a selective niche for increasingly sophisticated forms of shared intentionality (Tomasello & Gonzalez-Cabrera, 2017).^16^ In this context, shared intentionality would have co-opted the normative weight of great apes’ instrumental rational thought, facilitating the sharing of normative intentional mental states to support this form of high-risk collective action by reducing uncertainty about the actions of other group members. By reducing uncertainty, convergence on the Pareto efficient equilibrium would become more likely (i.e., no individual hunter could be better off without making at least one other worse off). This, in turn, would have split hominin instrumental reasoning capacities into individual and social subsystems. But since individual and social forms of instrumental reasoning would eventually provide conflicting policy advises (i.e., policies that maximize individual vs. shared goals), the evolutionary process likely required mutually adjusted changes in both capacities to reach the desired equilibrium.

Sterelny (2021) has argued against the view proposed here that normative thought was *not necessary* for early hominin collective hunting. He argues that since these activities involved a form of immediate return mutualism (see also Tomasello, 2016; Tomasello et al., 2012), norms were not necessary to control cheaters, including those who monopolize the whole profit of the collective enterprise and free-riders who simply increase the marginal cost of cooperation. In a situation of immediate return mutualism, he argues, foragers had little incentive to cheat, so cooperation would have been driven by purely individual instrumental reasons.

However, on a shared intentionality view, the capacity to represent and execute social norms initially evolved as a mechanism for solving *coordination problems*, thus *generating the profits of cooperation* (Calcott, 2008; Warneken, 2018), rather than as a cheating control mechanism.^17^ Coordination problems were multiple and complex for foragers without full-blown language; ancestral foragers had to converge on foraging strategy (e.g., whether they will hunt rather than gather), on defense policy (e.g., whether they will fight or fly from dangerous predators, whether they will prioritize the defense of the carcass over the safety of a hunting partner), and also on effort levels (e.g., time invested, foraging range willing to cover, risk exposure).

Moreover, getting access to animal carcasses is prone to coordination failures since outcomes are dictated by risk dominance rather than payoff dominance.^18^ Scavenging, hunting, or even gathering in the open are dangerous activities for hominins who lacked the speed, strength, natural weapons and defenses present in other species (Lieberman, 2018; Lieberman et al., 2009). Scavenging and hunting are also a risky investment. Success rates for big game hunting among Hadza are very low with extreme variance on returns (Hawkes et al., 1991), and although persistence hunters might be more successful, it requires tracking prey for long distances (27.8 km on average) and outcompete dangerous carnivores (Liebenberg, 2006). Thus, coordination problems were central to ancestral foragers and so too the need to manage coordination failures even if we assume that the threat of cheating was negligible.

If the above argument is correct, then, at some point in hominin history, shared goals and commitments would have been important to stabilize the generation (rather than the distribution) of the profits of cooperative foraging. Once shared goals are available, instrumental rationality would search in a different policy space: instrumental reasoning would become social instrumental reasoning, selecting policies aimed at socially optimal equilibria by co-opting the same phylogenetically old mechanisms discussed in Sect. 2.

Commitment to those policies would make instrumental sense too. For in a bipedal species like us, foraging not only have multiple equilibria (e.g., multiple foraging strategies, only some of which are collective) but also collectively optimal outcomes are risk dominated (e.g., in scavenging and hunting). Shared goals improve convergence on socially optimal equilibria in social decision making, and mutual commitment to those is necessary when aiming for optimal, but risky, foraging outcomes. In other words, shared intentionality would reduce strategic uncertainty over the actions of others making the socially optimal, Pareto-dominant option the focal point of equilibrium selection in foraging-related coordination games.

More importantly, social instrumental reasoning (i.e., instrumental reasoning that selects policies aimed at socially optimal equilibria) would have enabled agents to entertain shared normative intentional mental states that are *generalizable*, *intrinsically motivating*, and which *engender corrective attitudes*. First, it would have made possible to entertain shared normative intentional mental states of the form “We ought to φ.” These states are normative to the extent that they are the result of instrumental reasoning. But they are *generalizable* because such normative intentional mental states are represented from a shared perspective, which supports different degrees of abstraction from one’s egocentric perspective. An important driver of the expansion of social norms over the gradient of generalizability was likely the increasing demands on cooperation (including the solution of increasingly complex coordination problems) in the human lineage. For example, it may be that the scope of normative intentional mental states initially included only those who played a particular role in the group (e.g., the members of evanescent ancestral forager bands), with some of these norms (or versions of them) extending perhaps later to everyone in the camp, across clans in segmented societies, or perhaps even the whole ethnolinguistic communities, as a function of the fitness benefits generated through progressively larger organized collective action. The higher the demands for cooperation on human groups, the higher the degree of generalizability that is necessary for social norms to coordinate action that effectively increases cooperative profits.

Second, social instrumental reasoning enabled agents to entertain shared normative intentional mental states that are intrinsically motivating. In the shared intentionality approach, mental states such as goals are joint and shared because intense selection for cooperation in our lineage has made these activities intrinsically socially rewarding (Tomasello & Gonzalez-Cabrera, 2017). Similarly, under a shared intentionality view of the representation of social norms, it matters to the agent whether others in our social network think we ought to comply with a behavioral policy (for an extensive discussion of this feature of norm compliance, see Bicchieri, 2006, 2017).

Shared instrumental rationality not only motivates social norm compliance via intrinsic social reward but also by facilitating their *internalization*. Intentional normative mental states are internalized when complying with them becomes intrinsically rewarding regardless of social and individual incentives, i.e., when acting according to a norm becomes an end in itself rather than merely a means for achieving a certain (individual or social) goal or avoiding extrinsic punishment. This can make norm compliance somewhat automatic or instinctive. Yet, intrinsic motivation of this kind is not overriding. If the intrinsic reward of norm compliance is high enough, the subjective cost of violating the norm becomes higher than the perceived material benefit of its violation. But if the costs of compliance are too high, we should expect norms to be violated. On this view, norms can be seen as an argument in the utility function that each individual maximizes (Gintis, 2003).

Reinforcement learning of the kind described in Sect. 2 would have played an important role here. A social instrumentally rational agent can find a certain policy such as “Everyone in the band must help defend the carcass from other carnivores” or “Meat and honey must be widely shared by everyone in the band” to be instrumentally rational because it leads to stable returns from cooperation. This normative intentional mental state can be subsequently internalized because complying with its policy maximizes rewards and minimizes punishments, which can be both social (e.g., improving one’s reputation as a partner while reducing the risks of retaliation) and non-social (e.g., increasing the chances of obtaining meat and honey in the long run and reducing the risk of starvation). If a policy such as “Meat and honey ought to be shared with other band members” leads to positive outcomes, such as stabilizing cooperative foraging in contexts in which this practice is crucial for survival, complying with this policy will become a habit.^19^ By making it habitual, the agent could offload cognitive computation from model-based to model-free systems, encoding normative attitudes without the need of explicitly representing the norm.

Formal models suggest that an increasing capacity for social norm internalization evolves under a wide range of conditions involving cooperation to overcome environmental challenges and conflicts with neighboring groups assuming that a capacity to learn social norms by reinforcement is in place (Gavrilets & Richerson, 2017). Instrumentally rational agents (in both the individual and social sense) can develop preferences for partners who are intrinsically motivated to comply with a policy when successful cooperation depends on complying with it. For example, when food sharing is essential for cooperation and agents are able to track the mental states of others, it is instrumentally rational for them to prefer partners for whom complying with the policy “I ought to share meat and honey with other band members” is intrinsically rewarding (for a similar argument, see Stanford, 2018). This eventually could have led to the covariance between displays of trustworthiness and preferences for trustworthy partners, creating conditions for runaway social selection (Nesse, 2007). Certainly, signaling intrinsic motivation to others is difficult since motivational states are not salient, and thus must be tied to costly signals to be reliable. This is true even when language helped to make normative mental states more salient and publicly accessible.^20^ For this reason, signaling intrinsic motivation to comply with a shared normative intentional mental state often goes along with displaying corrective attitudes that are often costly and difficult to fake.

Thus, third, social instrumental reasoning could have enabled agents to entertain shared normative intentional mental states that generate *corrective attitudes*, as they are often part of the package deal of cooperation. Modeling and experimental work shows that norms evolve more easily and have larger effects on behavior if groups promote punishment for norm violators (Boyd & Richerson, 1992; Fehr & Fischbacher, 2004). Some corrective attitudes arguably played a key role in maintaining collaborative foraging practices in early humans (Boehm, 1999), which are thought to be supported by human-unique capacities for shared intentionality (Tomasello et al., 2012). These attitudes likely played a key role in reducing the threat of *free-riders*—those who benefit from cooperation but who do not pay the cost of it. But early hominins likely foraged in small groups in which free-riders were easily detectable. If collaborative foraging was crucial for survival and free-riding compromised it, instrumentally rational agents should have excluded those individuals from future cooperative interactions. Ostracizing free-riders in this way would be as instrumentally reasonable for agents (in both the individual and social sense) as is cooperating. Increasingly costly forms of punishment could have also emerged in those environments via reputation, since agents increase their fitness when decisions are based on reputation from punitive instead of cooperative actions (dos Santos & Wedekind, 2015). Other policies may perhaps engender costly but less harsh corrective attitudes for different reasons, including corrective behaviors in contexts other than collective foraging, such as teaching.

To sum up the discussion in this section, since shared intentionality and instrumental reasoning influenced each other’s evolution, these traits mutually adapted to each other. But if agents are able to entertain shared normative intentional mental states that are generalizable, intrinsically motivating, and which engender corrective attitudes, they are able to represent social norms (as defined in Sect. 1). This leads us to a substantial claim about evolutionary dynamics. As I have argued, the capacity to represent, endorse, and enforce social norms entails the capacity for normative guidance. Therefore, the capacity for normative guidance was the result of the coadaptation of shared intentionality and instrumental reasoning.

## Conclusion

In this paper, I have tried to contribute to the existing literature on normative cognition by providing a lineage explanation of human social norm psychology. Building upon previous theoretical approaches (Sripada & Stich, 2007), social norms are represented in this view by normative mental states that are characterized by their generalizability, intrinsic motivation, and corrective attitudes they engender. Based on this view, I have provided a lineage explanation of our distinctive capacity for normative guidance, i.e., our capacity to represent, endorse, and enforce social norms (Kitcher, 2011).

The above conclusions should, however, be interpreted with caution. Although perhaps useful for phylogenetic purposes, the proposed class of social norms is rather general and abstract. Much more research has to be carried out to flesh out the connection between the proposed psychology of social norms and, for example, moral norms. I have also assumed that human cognition is characterized by the normative mental states that mediate instrumental reasoning in non-human animals, especially our great ape ancestors. Although there seems to be good evidence supporting this claim (Camp & Shupe, 2017; Völter & Call, 2017), further work should aim at understanding better the scope and phylogenetic depth of this metarepresentational capacity. Similarly, future research should look more closely into the algorithmic nature of this reasoning capacity and the extent to which it can be captured by reinforcement learning models of cognitive control.

Finally, the proposed view is committed to a specific lineage trajectory of differentiation of normative guidance, which places its origins alongside those of shared intentionality—allegedly after the split between humans and apes (Hawkes, 2012; Tomasello et al., 2012; Tomasello & Gonzalez-Cabrera, 2017). On this view, species lacking these skills should not be expected to display normative guidance, which seems supported by available evidence (although for a view of potential precursors of social norms in great apes, see von Rohr et al., 2011, 2012, 2015). Tradeoffs between individual and social forms of instrumental rationality that tip the balance of norm compliance are also expected. Agents facing these tradeoffs should generally reach responses that resemble a Pareto efficient equilibrium in which individual instrumental rationality cannot profitably deviate given the policies chosen by the normative guidance system, and vice versa. Thus, departures of individual instrumental rationality when joining or sharing others’ goals should not generate normative regret, while departures leading to this form of regret should be associated to social norm representations that score high along the motivational and corrective axis of the agent’s representational space. More research is needed to test these hypotheses.

## Footnotes

1 Collective dilemmas include collective action problems, coordination problems, prisoner’s dilemmas, and other situations in which the aggregation of seemingly rational individual decision-making and action does not guarantee the optimal outcome for the group.

2 The fact that the representation of social norms has a motivational content deserves a more detailed discussion that goes beyond the scope of this paper. However, this feature of the approach should not come as a surprise. As Millikan (1996) has pointed out, inner representations of social roles, for instance, can have both descriptive and imperative content. This dual content is implicit in algorithmic views of reinforcement learning and goal-directed behavioral control, in which cognitive systems not only represent behavioral policies but also act on them.

3 To act in accordance with norms one can be both intrinsically and extrinsically motivated at the same time, as behavior can be motivated by many different goals and motivations at the same time. We can help someone because we care for the other person, and doing so is therefore intrinsically rewarding, while also enjoying the gratitude of others in response to this helping as an extrinsic reward.

4 In such a case, the space will have a topological structure. This is a common feature of conceptual spaces (Gärdenfors, 2000; for a more detailed discussion, see Mormann, 2021). The approach cannot, then, be simply dismissed on the grounds that the conceptual space at hand lack a metric and thus cannot be used to represent social norms.

5 Other approaches such as the assessor teaching model see normative though as an adaptation for cultural transmission but without linking it to model-based control (Castro et al., 2019, 2021; Castro & Toro, 2004). To my knowledge, this was the first approach linking the explicit transmission of complex cultural skills to normative thought.

6 Model-based motor control may not be the same as instrumental reasoning, but while skilled action might not be preceded by conscious deliberation, it might have been important when trial and error was costly but still informative for the learner. This especially important when considering explicit teaching. The transmission of the Acheulean handaxes manufacturing skills on Birch’s (2021) account, for instance, requires identifying an apprentice error, and responding to that error with appropriate corrective interventions that improve the apprentice’s performance.

7 An organism could be appropriately described as instrumentally rational even if it is not aware of the underlying inferential process involved in behavioral control. Although reasoning is often understood as involving self-awareness, this condition can be relaxed to allow cases of inference without reckoning (Siegel, 2019). For the sake of clarity, I will refer to instrumental reasoning as requiring self-awareness.

8 The connection between self-conscious reflective capacities and normativity has been explored by philosophers such as Korsgaard (1996). On this approach, reflective capacities help us to decouple agency from primary behavioral impulses, which forces the agent to exert a complex form of normative self-governance. The view I defend similarly preserves a special role for conscious deliberation in normative thought.

9 As pointed out by one of the reviewers, human reasoning capacities could also be shaped by vigilance towards communicated content as argued by Mercier & Sperber (2017). However, the reasoning capacities discussed in this literature are closely linked to testimony and relatively complex linguistic skills, which I assume played a late role in human evolution. Giving the conventional nature of linguistic communication, a baseline form of social norm psychology was likely a precondition for fully linguistic agents.

10 I use the term ‘shared intentionality’ to refer to the meshing of intentional mental states that supports joint activity and that is somewhat captured in ordinary language through the use of the plural subject ‘we’ (for similar definitions in the psychological literature, see Call, 2009; Tomasello et al., 2005; Tomasello & Carpenter, 2007).

11 The terms ‘joining’ and ‘sharing’ are often indistinctively used in the literature. However, the former can be used to refer to the process of engaging in another individual’s intentional mental state, while the latter can be used to specify the skills that help others to join one’s mental states (e.g., by signaling or communicating one’s states to invite others to join them).

12 Normative states in the we-mode do not need to be collective mental states as perhaps suggested by expressions of the form “We ought to φ.” For these states can be represented in such a mode when the propositional attitude is in I-mode but its propositional content is in we-mode, as in “I think we ought to φ.”

13 Beyond observational reports (Gómez, 2010; Pika & Zuberbühler, 2008; Tanner & Byrne, 2010), experimental evidence of reengagement has been reported in the literature (Heesen et al., 2020). However, preferences for social activities are not themselves evidence of commitment, which would be more suggestive of normative guidance.

14 Chimpanzees are also known to incur costs to watch the punishment of antisocial individuals who have directly wrong them (Mendes et al., 2018), but they are equally motivated to watch the punishment of prosocial and antisocial individuals when observing third parties interacting. Thus, the group concern interpretation should be taken with caution.

15 The above argument does not rule out the possibility that other less cognitively and motivationally demanding forms of shared intentionality, such as joint attention, are present in great apes. However, these forms of shared intentionality are less central for the psychology of social norms than shared goals and commitments.

16 Although it is usually agreed that meat was an important part of Plio-Pleistocene hominin diets, opportunistic strategies such as passive scavenging cannot afford significant meat yields (Domínguez-Rodrigo, 2002). Moreover, there is evidence of 1.7 mya Acheulean technology associated with butchery of carcasses (Diez-Martín et al., 2016). The killing of these animals was likely the result of ambush as some have suggested (Bunn & Pickering, 2010; Pickering, 2013; Sterelny, 2021) since targeting big game is dangerous, even when the prey is vulnerable. For example, large antelopes are still a serious threat when they are injured. Also, foragers have to compete with other carnivores that are faster, well adapted for predatory, and in need of meat to survive. Thus, unlike the kind of spontaneous hunting of monkeys we see in chimpanzees, this kind of hominin hunting required manufacturing specialized tools, carrying them for a foreseeable purpose, moving along in groups, and even some communication skills to coordinate the ambush.

17 The role of norms as coordination devices has been highlighted by others (e.g., Lewis, 1969; Ostrom, 2000; Ullmann-Margalit, 1977). A well-known empirical method to measure social norms employs coordination games to elicit those norms under the assumption that subjects use their normative expectations to solve these games (Fallucchi & Nosenzo, 2022; Krupka & Weber, 2013). In the proposed view, however, this does not mean that coordination is the only (or the defining) function of social norms but that our capacity to represent and be guided by them initially emerged in the hominin lineage as a solution to coordination problems.

18 Experimental evidence shows that pareto-dominant outcomes do not act as focal points in coordination games (Cooper et al., 1992; Devetag & Ortmann, 2007; Van Huyck et al., 1990). Evolutionary approaches to game theory also suggest that the risk dominant equilibrium strategy is more likely to occur than the payoff dominant equilibrium (Kandori et al., 1993; Nax & Newton, 2019; Sawa & Wu, 2018; Young, 1993).

19 While traditional model-free accounts of habitual norm compliance cannot explain the persistence of norm compliance when environmental contingencies cease to reinforce the habit, tutor accounts of the basal ganglia explain habitual behavior in ways that are significantly entrenched against changes in reward contingencies. This is so because when the basal ganglia is involved in decision-making in a novel context, this group of structures slowly train cortical and subcortical mechanisms, eventually transferring the relevant decision-making to them as the decisions become habitual (Huang, 2017).

20 Language would have made behavioral policies increasingly socially salient in a similar way as metarepresentational capacities facilitated conscious forms of normative thought and reasoning. This makes the role of language in the emergence of normative thinking rather late and circumscribed as opposed to counterfactual genealogies of morality that begin with fully linguistic agents and the necessity to establish one credibility as a cooperative partner (Pettit, 2018).

## Glossary

Coadaptation: Process in which interacting traits undergo natural selection together in response to the same selective pressure or when selective pressures alter one trait and consequently change the interactive feature.
Goal-directed control: A form of instrumental control that relies on a model-based computational approach that represents the goals of the agent and the contingent relationship between behaviors and outcomes.
Lineage explanation: An explanation that aims to provide a tentative sequence of changes that makes increasingly plausible the emergence of a certain feature from a baseline of preexisting mechanisms within a biological lineage.
Model-based controller: A system that takes decisions based upon an internal model of the agent’s goals and the contingent relationship between actions and outcomes, which is built through reinforcement learning processes.
Policy: A mapping from the learning agent’s perceived states of the environment to actions to be taken when in those states.
Policy gradient method: A reinforcement learning method that optimizes parameterized policies with respect to long-term cumulative reward.
Reinforcement learning: A branch of machine learning concerned with training agents to operate in an environment in order to maximize their cumulative reward in the pursuit of some goals.
Shared intentionality: The meshing of intentional mental states that supports joint activity and that is somewhat captured in ordinary language through the use of the plural subject ‘we.’
Social norm representation: A special type of normative mental states that are defined by a gradient of generalizability, intrinsic motivation, and corrective attitudes.
